# Islet Autoantibody Detection by Electrochemiluminescence (ECL) Assay

**DOI:** 10.4172/2155-9899.1000531

**Published:** 2017-12-11

**Authors:** Yong Gu, Zhiyuan Zhao, Hilary High, Tao Yang, Liping Yu

**Affiliations:** 1 Barbara Davis Center for Childhood Diabetes, University of Colorado Denver, Aurora, CO, USA; 2 Department of Endocrinology, First Affiliated Hospital of Nanjing Medical University, Nanjing, Jiangsu, China

## Short-Communication

Islet autoantibodies, used as markers for the autoimmune destruction of insulin producing β cells, plays an essential role in predicting and diagnosing type 1 diabetes (T1D). For decades, mechanistic bioassays of islet autoantibodies (IAbs) have been gradually developed against a current panel of four major islet autoantigens, namely, insulin (IAA), glutamic acid decarboxylase (GADA), islet antigen 2 (IA-2A), and zinc transporter 8 (ZnT8A). In order to improve and standardize measurements of IAbs associated with T1D, the Islet Autoantibody Standardization Program (IASP) and Islet Autoantibody Harmonization Committee were established and put in great efforts since the year of 2000 and 2007, respectively, led by NIH/NIDDK, the Center for Disease Control and Prevention (CDC), and the Immunology of Diabetes Society (IDS) [1-4]. While many laboratories in IASP workshops had less than acceptable sensitivity or specificity, especially the insulin autoantibody assay. The reason may lie in the limited conformational epitope recognition of antigens by antibodies where antigens are bound to the solid phase in the traditional enzyme-linked immunosorbent assay (ELISA). Therefore, the radio-binding assay (RBA), a fluid phase assay using radiolabeled antigens to facilitate identification of immuno-reactive antigen regions, has been established as the current ‘gold’ standard assay for all four major IAbs. However, the works are still in progress, especially for the IAA assay, which is still problematic in most laboratories. Furthermore, the use of radiation limits the application in clinical lab and the single assay capacity limits routine screening in large scale population.

**Figure 1:**
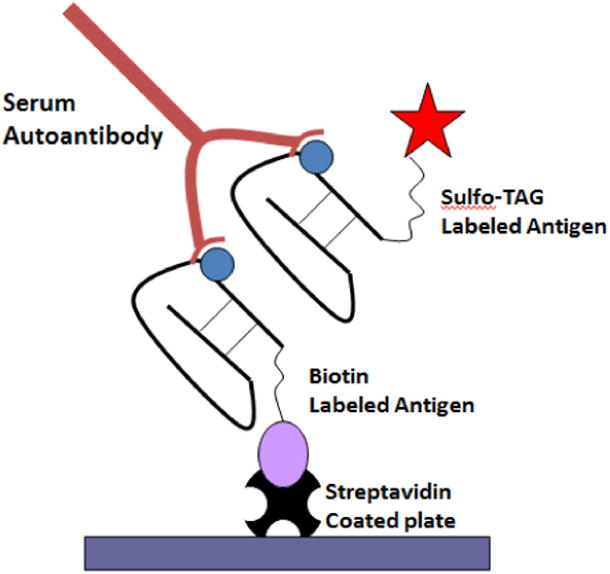
Electrochemiluminescence (ECL) Assay

Recently, we have developed a novel electrochemiluminescence (ECL) assay with higher sensitivity and specificity than the conventional ELISA and RBA, which is based on the principle of interactions for autoantibodies with antigen molecules in the nonradioactive liquid phase [5,6]. In the ECL assay, the IAbs in the serum bridge the Sulfo-TAG conjugated antigens to the biotinylated capture antigens, which are then captured on the solid phase of the streptavidin-coated plate as shown in Figure 1. The detection signals are from the Sulfo-TAG label found on antigen molecules bound to specific autoantibodies. As opposed to the conventional ELISA, the 2nd labeled antibody (labeled anti-human IgG antibody) is not used for detection. Using the 2nd antibody usually causes a very high background in autoantibody assays for human samples. In the issue of Methods in Molecular Biology, we described the methods of ECL IAA and GADA assays in detail [7]. To perform the assay, serum samples are mixed with both Sulfo-TAG conjugated and biotinylated capture antigen protein. After incubating overnight, the serum-antigen mixture is transferred onto a prepared streptavidin-coated plate and is incubated for another hour. The plates are then washed and counted on the MSD plate reader machine. The results are conveyed as an index, which was generated from internal standard positive and negative control serum samples.

IAbs serve as key risk markers for both enrollment criteria and follow-up end points in T1D prevention trials. An ongoing international clinical trial, The Environmental Determinants of Diabetes in the Young (TEDDY), depends on the accurate detection of IAbs to pinpoint the timing and appearance of the first islet autoantibody. This first islet autoantibody acts as a primary ‘seroconversion’ to mark the very beginning of islet autoimmunity and help to identify environmental triggers. A highly sensitive assay with a high disease specificity that accurately detects T1D relevant IAbs on the right time is important. The current standard RBA method, especially seen in the IAA assay, has not yet achieved a satisfactory level of sensitivity and specificity. Importantly, IAA has a high prevalence and is usually the first autoantibody to appear among young children [8,9]. A study using the Diabetes Autoimmunity Study in the Young (DAISY) cohort showed that the age of diagnosis in diabetes was strongly correlated with the age of appearance of the first autoantibody and the levels of IAA present [10]. Compared with standard RBAs, we have analyzed thousands of participants followed from birth with the new ECL assays in the clinical trials, DAISY and TEDDY and closely monitored the time of ‘seroconversion’ for IAb appearance for these two assay methods. ECL assays, especially the ECL-IAA assay, demonstrated more sensitive and were able to define the timing of the initial autoantibody appearance earlier than the RBAs. ECL-IAA antedated the onset of islet autoimmunity in young children by a mean of 2.3 years (range: 0.3-7.2 years) in the DAISY cohort [11]. The earlier identification of IAA among young children was validated from a later TEDDY study cohort (unpublished data). In the DAISY longitudinally follow up study, we analyzed 427 sequential samples from 63 pre-T1D children, who were closely followed to clinical T1D. Nearly all of these children (62/63) had IAA detected using the ECL assay years before disease onset. This includes the 10 children who were found to be completely IAA negative using the RBA during the whole follow-up period. Remarkably, 25% of the pre-diabetic samples which were positive for ECL-IAA were found to be negative for the RBA-IAA. A possible reason for increased sensitivity in the ECL assay is that the assay captures all of the immunoglobulin classes: IgG, IgM, IgA, and IgE. The traditional RBA or ELISA relies only on the detection of IgG.

On the other hand, the ECL assay has shown a superior advantage in discriminating against high disease specific IAbs from low-risk signals generated by RBAs [5,6,12]. Specificity of antibody measurement is commonly defined as the ability of an assay to score a positive result when the serum sample contains an antibody that can bind and/or neutralize the target protein. Disease specificity of IAbs refers to the high-affinity and high-risk shown with high disease predictive values. Non-disease specific IAbs are especially noticed in subjects who are only single IAb positive, usually single IAA or single GADA, and have never progressed to type 1 diabetes. Most of these have been found to be low-affinity and low-risk autoantibodies. During follow up testing, done within months to years, these autoantibodies were lost, behaving as ‘transient’ and biologically ‘false positive’. As previously proposed [5], these low affinity ‘single’ autoantibodies likely resulted from an immunization with a cross-reactive molecule, while the higher affinity, higher risk IAbs likely resulted from an immunization with the islet antigens themselves. In a study of DAISY children [5,6], we analyzed all pre-T1D children, who were followed to clinical diabetes, and all non-diabetic children who were persistently IAb positive, either having multiple or single autoantibodies positive. Almost all of the samples that were positive by RBA (IAA or GADA) were positive in the ECL assay for children with pre-T1D and for children with the presence of multiple IAbs. In contrast, only around 25% of the non-diabetic children with either a single IAA or a single GADA positive by RBA were also found to be positive by the ECL assay. The antibody affinity study was performed to compare IAA and GADA detected by the RBA, but differentiated as positive or negative by ECL assay. The results of the affinity analysis discovered that when IAA or GADA was detected by the RBA, but found negative in the ECL assay, there was a low affinity. In contrast, when either IAA or GADA were found to be positive by both the RBA and the ECL assay there was a high affinity. In our later validation study with a large TrialNet cohort called Pathway to Prevention, the same conclusions were made [12] using the characteristics of disease specificity for both ECL-IAA and ECL-GADA. The ECL assay and RBA were found to be consistent in pre-diabetic subjects and in the subjects with multiple IAbs. Only 24% of the single RBA-IAA (p<0.0001) and 46% of the single RBA-GADA (p<0.0001) were confirmed by the ECL-IAA and ECL-GADA assays, respectively. With prospective follow-up for subjects with a single IAA or GADA, 51% of RBA-IAA and 63% of RBA-GADA subjects that were not confirmed using ECL were found to have lost their IAbs and became IAb negative after the mean follow-up time of 2.4 years, behaving as a ‘transient’ IAb positive. The antibody affinity was further analyzed [13] in a sequential follow-up study (mean follow up time of 5.3 years) from a subset of subjects in the TrialNet Pathway to Prevention study. These subjects were persistent for single RBA-IAA or single RBA-GADA using the RBA and were either confirmed or not confirmed using the ECL assay. The subjects had either a single IAA or a single GADA confirmed positive by the ECL assay and were found to have high affinity autoantibodies already present at their very first initial visit. The affinity results stayed consistent over time. Similarly, those who were negative in the ECL assay showed lower affinity at the initial visit and the affinity stayed lower over time. There were no converting events from low to high or high to low affinity seen over the time of the study. Analyzing the metabolic data in the TrialNet study [14], subjects who had either a single IAA or GADA present in the RBA, but not confirmed in the ECL assay, showed no buildup of glycemia. In contrast, glycemia increased significantly in the ECL-positive single IAb participants and the glycemia increase was found comparably as seen in multiple IAbs positive subjects who were known to have a high risk for developing T1D. The results from the above studies clearly demonstrate that the ECL assay is able to separate between two types of IAb positivity generated by the RBA, high affinity and high risk from low affinity and the subjects having no or less disease risk. Furthermore, high risk T1D autoantibodies with high affinity are able to be identified in the very beginning of initial screening for the subjects with either a single IAA or a single GADA through the ECL assay. This provides a great benefit to clinically predict the risk of T1D development in the early stages with only a single IAb.

There are about 85%-90% of the children who develop T1D who have no family history. General population-based screening for the risk of T1D, especially in young children, becomes important and is needed [15-17]. To fit into the purpose of large scale screening for national clinical trials and for the general population, people are starting to seek credible multiplexed assays, combining multiple autoantibody assays together into one single test. With the platform set for single ECL assays, a multiplexed assay can be expanded upon. It can simultaneously determine up to 10 different autoantibodies in one single well with a tiny amount of serum. Currently, four IAbs including IAA, GADA, IA-2A and ZnT8A are equal in importance for predicting the risk of progression to T1D in both relatives of patients with T1D and the general population. The methods utilized for screening these 4 IAbs, using current single autoantibody measurements, are laborious and inefficient, especially for large scale population screening. Importantly, up to 40% of patients with T1D have an additional autoimmune condition and it is highly recommended to screen these related autoimmune diseases during T1D screening.

**Figure 2:**
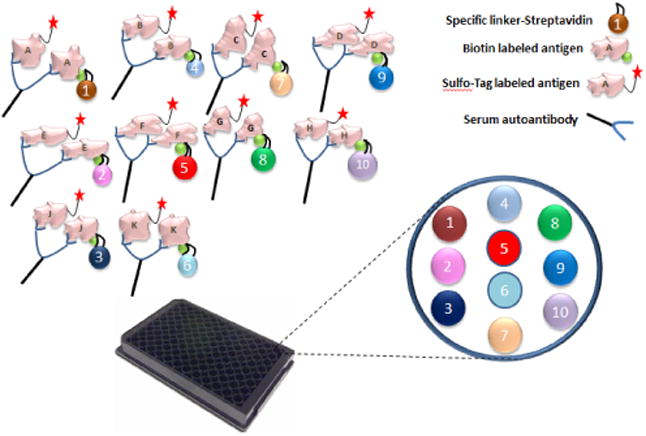
Multiplex ECL Assay

Unfortunately, currently there is no easy and inexpensive tool to screen for these conditions. Multiplex ECL assay is not only capable of combining 4 current major IAb assays into one, but they are also able to further combine more autoantibody assays together for other relevant autoimmune diseases. This makes it possible to efficiently conduct high throughput screening for multiple autoimmune diseases simultaneously. In the multiplex ECL assay, each antibody-antigen complex formed in the fluid-phase will be restrained to a specific linker source spot in the same well as shown in figure 2. The signal receiver on the plate reader machine is able to recognize the signals from 10 different sources of spots. For long-term studies using multiplex ECL assay, it is recommended that the same linker be used for the same autoantibody assay to keep the assay consistent. There are 4 main advantages when using a multiplex assay, compared to the current single radioassay: 1) higher throughput capacity: one lab technician is able to complete multiple autoantibody tests in a single assay; 2) lower volume of serum: the multiplex assay only needs 5 μl per well to test multiple autoantibodies (up to 10) compared to the single assay which uses 5 μl to test only one autoantibody; 3) lower cost: depending on the number of the multiplex, the multiplex assay will reduce the cost by up to 70% as compared to the total cost for correspondingly single assays; 4) non-radioactive: as the multiplex ECL-assay does not require radioactivity, we envision increased access to autoantibody measurements with this methodology across academic and commercial laboratories.

In conclusion, IAbs are currently the best biomarkers for T1D and have contributed to the understanding of the natural history of the T1D autoimmune process and the prediction of risk in both first-degree relatives of T1D patients and the general population. Recently developed Electrochemiluminescence (ECL) assays for IAbs are more sensitive than the current ‘gold’ standard RBAs, able to identify the initiation of islet autoimmunity earlier in life among high-risk children before clinical onset of diabetes, and are more disease specific because they are able to discriminate high-affinity, high-risk diabetes specific IAbs from low-affinity, low-risk autoantibodies. There are still many challenges and there is still room for improvement, especially in assessing the general implementation of methods for the assay across different laboratories and having these methods be standardized. With the improvement of comparative assessments of results, multiplexed ECL-assays combining multiple antibody assays into one single well will be a useful tool for general population screening.
